# Healthcare workers and adult patients preferences of hospital built environment. Survey in ordinary surgery and medical oncology ward at the Italian National Oncology Institute

**DOI:** 10.3389/frhs.2025.1546103

**Published:** 2025-04-07

**Authors:** Andrea Brambilla, Gabriele Mario Perotti, Valentina Villa, Isabella Nuvolari-Duodo, Antonio Triarico, Carlo Nicora, Stefano Capolongo

**Affiliations:** ^1^ Design and Health Lab, Department of Architecture, Built Environment and Construction Engineering (DABC), Politecnico di Milano, Milan, Italy; ^2^ Agenzia Regionale Emergenza Urgenza (AREU), Lombardy Region Regional Agency for Emergency and Urgency, Milan, Italy; ^3^ Foundation IRCCS, National Oncology Institute, Milan, Italy

**Keywords:** oncological setting, inpatient room, healthy environment, users involvement, evidence based design, healthcare infrastructure

## Abstract

**Background:**

Experience is an important factor in hospitalisation and treatment processes, especially in oncology. The preferences of patients and health workers have recently been increasingly considered as key elements for supporting clinical and organisational performances. The relationship between staff and patients preferences and the quality of hospital built environment is also an important aspect but it is still underexplored in the scientific literature.

**Aim:**

The study aims to understand both qualitatively and quantitatively how the hospital built environment influences the well-being of patients and staff in ordinary surgery and medical oncology ward of a national institute for oncology in Northen Italy.

**Methods:**

The research was carried out according to the following methodological sequence: (i) identification of the target and setting; (ii) elaboration of a questionnaire with 22 items; (iii) administration of the questionnaire on a sample of patients and health professionals; (iv) data collection in a dedicated database; (v) data analysis and interpretation.

**Results:**

A total of 521 adult oncology patients and 311 health workers participated in the study. The findings highlight differences and similarities of preferences among patients and staff regarding built environment features that are reported in scientific literature. For example, patients shows limited interest in the possibility of having a single room (only 31% report it as very important), while from the staff point of view, there is a predominantly importance-oriented distribution with 51% and 83% of the respondents that consider this to be of great relevance respectively for their practice and for the patient experience.

**Conclusion:**

The findings underline the importance of considering the perspectives of oncological patients and healthcare workers in the assessment of oncology wards for future evidence-based hospital design.

## 1 Introduction

### 1.1 The challenge of user empowerment in oncological setting

Oncological disease represents one of the most complex and demanding challenges in global health. Characterized by a wide range of types, stages and treatments, oncology requires a multidisciplinary approach and a wide range of resources to ensure comprehensive and effective patient care (1).

First, there is the diversity of tumor types which imply in-depth and up-to-date knowledge on the part of healthcare professionals. Secondly, cancer disease often involves multiple body systems and organs, making treatment and symptom management particularly complex. In addition, cancer disease is characterized by several complications that may include hypertension, diabetes, heart disease and psychological disorders (1).

As well as cancer patients, healthcare professionals face a number of complexities managing the clinical situation of patients, communicating with them and managing workload. The current and predicted future shortage of physicians and nurses is also problematic: it has been projected that in the United States by 2025 there will be a shortage of between 124,000 and 160,000 full-time physicians, other than a potential shortage of more than 1 million nurses by 2020 (2).

Within this context, there is a growing interest in the role of the physical environment in determining the well-being of users and health professionals. In fact, the built space is a crucial element for cancer patients and health professionals, as work in this context is particularly stressful and hospitalisations are longer than average (3). There is also a need for places that contemplate privacy, considering the frequency of communication of personal data and sensitive matters.

In this regards, it has been pointed out that cancer patients often express a desire to be more involved in their own care (4). However, in Italy, patient awareness and participation in the care process is still limited. Many authors suggest that the entire medical team should involve the patient as an expert and consider their experience and knowledge as an integral part of the care process, stressing the concept of patient empowerment (4, 5) as a process through which people can gain more control over decisions and actions concerning their health.

One of the fundamental characteristics of empowerment is that it puts patient participation at the center (4). Participation can happen in treatment decisions, involvement in service development, integration of service evaluations from the patient's perspective, participation in training and education, and active participation in research activities (6). This study is positioned in a stream of research that consider the process of healthcare user empowerment very relevant also for the design of hospital spaces.

### 1.2 The impact of the built environment on users well-being

A widley known approach to deal with the physical setting in healthcare environments is the Evidence-Based Design (EBD). Studies in this field demonstrates the significant impact of the built environment on users (7). EBD is an approach that emphasizes the use of acquired data to influence the design process in hospitals: it measures the physical and psychological effects of the environment. The solutions derived from the results can address the real needs of each hospital and improve technological best practices to reduce infections, stress and improve patient comfort (8). Humanisation, thus, acquires a therapeutic purpose: the healthcare facility must be able to support the individual during the experience of illness, facilitating the process of psychological adaptation (9). For example, several studies have shown that natural light can help reduce depression, relieve fatigue and regulate circadian rhythms (10). It is also associated with increased concentration, satisfaction and positivity, as it can influence the release of serotonin (11). It is important to consider natural light exposure in healthcare environments, providing windows in patient rooms to maximise sun exposure and ensuring adequate access to natural light in staff rooms (10). There is preliminary evidence showing light, noise and blue spaces potential roles in the survivorship experience among cancer patients (12).

Another element that contributes to people's well-being is nature or the creation of environments that evoke it. Roger Ulrich, through his research, has shown that even a passive view of nature through windows or simply through a painting can promote positive moods or reduce stress (13). Ulrich also highlighted how viewing through a window can influence recovery from surgery by reducing the length of hospital stay. Several studies have shown that exposure to nature can reduce heart rate, muscle tension and blood pressure (14).

In conclusion, the presence of nature in healthcare environments can improve people's emotional and physical wellbeing, offering them an opportunity to connect with the natural environment that can promote healing and recovery. Cancer and its treatment imposes a certain vulnerability and potential lack of control. Empowering patients with options in the treatment space, including capacity to control ambient and furniture conditions, play a role in balancing such potential feelings of lack of control (15).

Lastly, single rooms can improve the overall patient experience and also help reduce the risk of infection (3, 16, 17). Not only they provide greater comfort and privacy for patients, but also represent a protective measure against the spread of infections, which is particularly important for patients with compromised immune systems. This spatial arrangement allows patients to be isolated more effectively, limiting exposure to pathogens and contributing to the prevention of nosocomial infections.

### 1.3 Research gap and aim of the study

It is noted that, while it is possible to take into account the organizational needs of medical management during the design phase, it is more difficult to represent the needs and preferences of patients and workers (18). User-centered care positions the patient and staff at the core and points up satisfying their needs, preferences and values. Although widely recognized as necessary, this approach is not universally implemented. Hospitals are built to meet the expectations and perspectives of staff and policymakers while incorporating patients' views is vital when designing a hospital (19). In the context of oncological patients their preferences are rarely asked during significant decision-making processes. Moreover, there are only few studies concerning preferences on aspects related to the built environment, apart from specific cases related to pediatric patients or the specificity of green spaces (20–22). Indeed, the literature shows a dearth of specific studies on the perceptions of healthcare professionals and practitioners in oncology hospital environments as the science of how to change the built environment to improve cancer health outcomes is still in its infancy with several unanswered questions (23).

Against this background, an in-depth qualitative and quantitative study has been conducted in a specific hospital environment. The aim was to further explore how the hospital environment characteristics are perceived by patients and healthcare workers to support wellbeing and operational performance. The investigation has been conducted thanks to the availability of a primary healthcare institute for cancer treatment and research in northern Italy, specifically the National Cancer Institute in Milan. The results of this case study could provide a general approach to the investigation of oncological hospital space, important evidence-based insights to improve the design and management of hospital spaces, and eventually contribute to the overall quality of healthcare journey experience. Given that most of the literature on physical environments in hospitals are primarily related to user satisfaction with service delivery, the questionnaire collects users' perspectives on design-related aspects with the potential to influence their interaction with the environment and increase practitioner participation in healthcare design.

## 2 Methodology

The present cross-sectional observational study uses a mixed methodology, combining quantitative and qualitative approaches to obtain information on the relationship between the physical spatial characteristics of hospital wards and the perceptions of adult patients and healthcare professionals through a structured survey submitted to a sample of hospital users in a case study of northern Italy.

**Figure 1:**
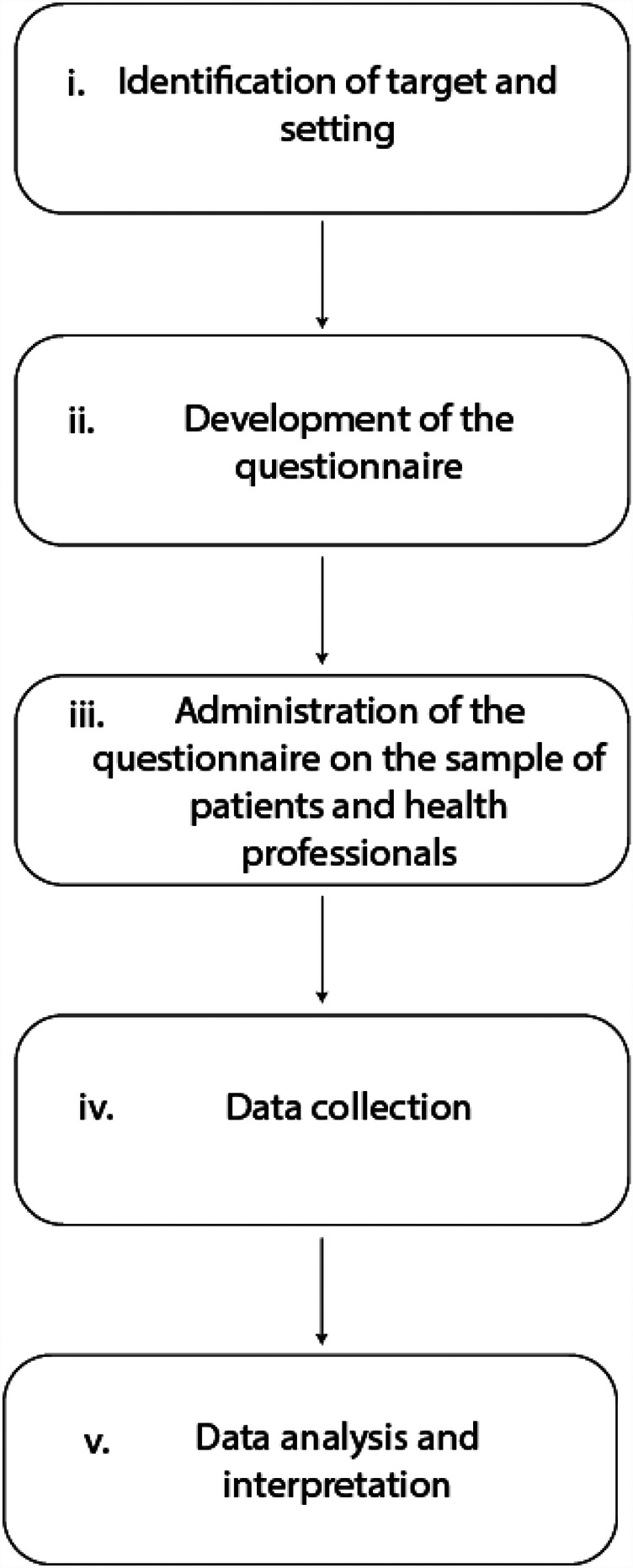
Flowchart of methodological steps.

As represented in Figure 1, the research was conducted following the methodological sequence reported below:

(i) Identification of the target and the setting; (ii) development of the questionnaire; (iii) administration of the questionnaire on the sample of patients and health professionals; (iv) data collection in a dedicated database; (v) data analysis and interpretation.

### 2.1 Identification of target and setting

The study is represented by a questionnaire survey addressed to all adult cancer patients admitted to ordinary medical and surgery ward in the National Oncology Institute of Milan, Italy, a primary healthcare center for cancer care and advanced research accredited to the national healthcare system (Istituto Nazionale dei Tumori—INT).

The National Cancer Institute (INT) is a Scientific Institute for Hospitalization and Treatment (IRCCS in Italian Legislation) in the oncology area with 383 ordinary beds, 47 day hospital/day surgery beds and 20 beds of complex outpatient macro-activity/low intensity surgery.

The Foundation's main covers approximately 86,000 square meters and houses all the departments dedicated to clinical activity, part of the research departments, the administrative offices and a teaching area.

The complex provides an average of 12,000 admissions/year in ordinary inpatient care, 4,000 admissions in day hospital care, and approximately 1,200,000 outpatient services (specialist visits and instrumental diagnostics).

The institute has a total of around 1,600 employees, of whom 350 are in the management area and 450 in the nursing area.

**Figure 2:**
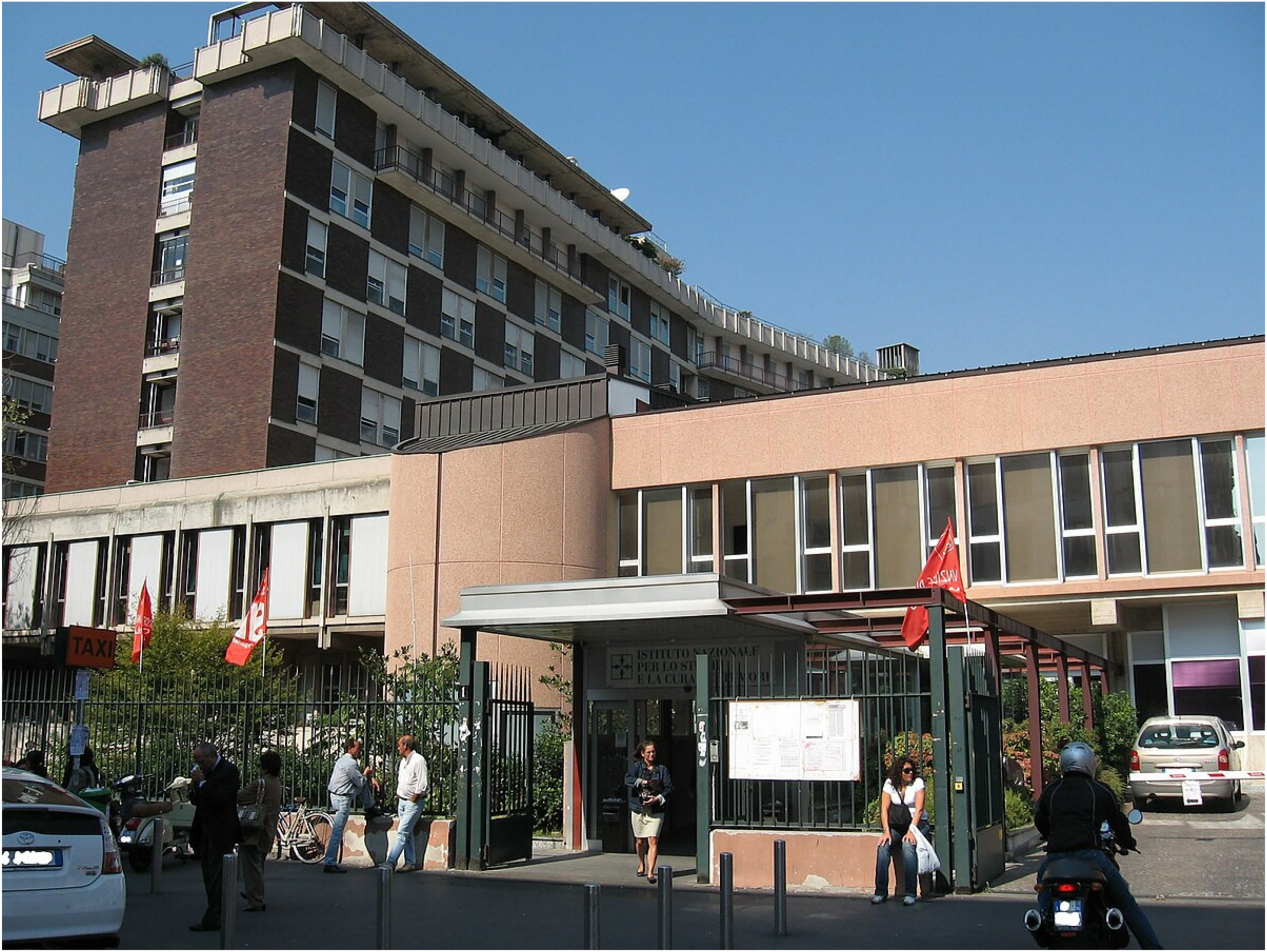
National Oncology Institute of Milan, Italy. Reproduced with permission from “Istituto Nazionale per lo Studio e la Cura dei Tumori, Milano” by Gia.cossa, licensed under CC BY-SA 4.0.

The hospital facility, shown in Figure 2, in its current state it is composed of several connected buildings. Over the years has undergone numerous interventions, some of considerable importance, with additions, demolitions and modifications: in general, the in-patient wards have various types of layouts, with one, two and even three-bed rooms, almost all with en-suite bathrooms.

The choice of this target was determined by the possibility of having an adequate number of adult patients accessing a single-specialist facility for the same reason and health needs, and therefore having an homogeneous sample of this patient cohort. The choice of the ordinary hospitalization regime was determined by the need to identify the type of adult patients who spends the majority of their time in an in-patient room, and therefore with a specific interest in the usability and quality of the layout of the room itself. No restrictions have been included in the selection of healthcare workers sample. The pediatric departments have been excluded due to their specific needs but could be included in further studies.

### 2.2 Development of the questionnaire

The questionnaire was submitted to users in paper format, on a single A4-size sheet, printed double-sided. In the initial part, there was a brief presentation of the study, as well as the guarantee of anonymisation of the data collected. There were therefore some fields to be filled textually, such as the fields relating to the compiler's age, date of completion and province of birth, while otheres where it is necessary to tick the box, for example for the compliler's gender and above all for the individual technical items proposed. The limited literature on the subject does not allow the use of structured or validated scales to measure the preference of variables relating to the built environment. Neverheless each included built environment characteristics has been found as relevant in recent scientific studies or literature reviews on the topic (12, 15, 24).

Therefore, a collection of questions regarding the preference of certain variables was chosen on the basis of a likert scale from 1 to 5. The evaluation of the technical items was prepared by means of a five-level scale of appreciation/importance given by the users, where 1 represents the absence of importance and indifference to the presence of the tested characteristic, 3 represents an intermediate solution corresponding to “quite important” or of medium interest on the part of the users, while 5 represents the highest degree of importance “very important” and the highest level of interest on the part of the users.

The questionnaire items were identified on the basis of a review of the literature and relevant guidelines in the field. The purpose of the review was to identify design factors which:

- change the physical environments in healthcare facilities;
- influence users' perception and satisfaction with the physical environment;
- influence service delivery and clinical outcomes

During the pilot phase of the project, it was decided to investigate which physical environmental characteristics in hospital patient rooms contribute to patient well-being and are considered by patients to be most relevant. The choice of the 22 items to be proposed to the users was made in relation to the areas defined in the theoretical background relative to the design of the in-patient rooms, i.e., on the basis of the existence of evidence and/or studies documenting their importance and significance in terms of improving the quality of the in-patient stay, the operation of the rooms, the comfort and/or safety of the patient and the operators. The full list of items is provided in Table 1.

**Table 1:** Questionnaire's list of items.

N°	List of items
1	Have a single room, so that privacy is fully respected.
2	Have a two-bed room, so they can socialise to avoid the boredom of hospitalisation, and facilitate supervision.
3	In the case of a double room, how important is it to have a separation between the two beds by means of a curtain.
4	In the case of a double room, how important is the presence of a physical separation between the two beds through a movable wall.
5	Having a living area in the patient room so that the patient can relax without staying in bed.
6	Having the possibility for the patient to look out of the window while remaining in bed.
7	Have the windows of the in-patient rooms with a view of the surrounding nature (if the facility is located in the countryside) or of the surrounding buildings (if the facility is located in a city context).
8	Have an air-conditioning system in the room that the patient can regulate from their bed.
9	Having the possibility for the patient to adjust the room lighting from their bed.
10	Have a nurse call system accessible to the patient not only from the bed, but in every area of the room.
11	Provide a second bed or a bed chair for a possible patient partner/parent.
12	Positioning the bed in line with the door, so that it is also visible from the corridor and facilitates surveillance by the operators.
13	Presence of a video surveillance system connected to the guardhouse.
14	Have a private bathroom for the patient accessible from the room.
15	Have a bidet in the bathroom.
16	Room walls in soft, uniform colours.
17	Brightly coloured room walls.
18	Room walls decorated with natural landscapes, parks, mountain views, etc.
19	Presence in the room of services such as Wi-Fi connection, satellite TV.
20	In the case of double rooms, a dedicated TV in each patient bed equipped with headphones.
21	Presence of soundproofing in the room to exclude any noise from the ward corridor or neighbouring rooms.
22	Possibility for patients to be able to customise the furniture in their rooms by making small changes and/or enriching them with their own items.
23	Having the nursing room at the beginning of the ward.
24	Having the nursing room in the centre of the ward.

### 2.3 Administration of the questionnaire on the sample of patients and health professionals

The survey was carried out over a period of 1 month, starting on the 1st of January 2024, through the distribution of questionnaires in all the wards and services of the INT, with the exclusion of in-patient areas and services intended for pediatric patients: this type of patient was excluded in view of the particular nature of the needs expressed by this type of patient and the impossibility of comparing them with those of adult patients.

The questionnaire was given to the ward nurse coordinators who were instructed to hand in only one questionnaire even in the case of repeated admissions: participation in the study by patients was in any case presented as voluntary. The same questions were addressed to the operators and health professionals working at the INT, in order to collect their perceptions for 1 month, starting from the 1st of April 2024. With regard to the administration on the operators the methodology adopted is based on the exigency-performance approach, which, through the analysis of the characteristics of the operators and the activities carried out in their structures, leads to the definition of the exigency framework and the formulation of suggestions for the design of the spaces. Specifically, each question asked of the patients was divided into the following specific sub-questions:

(a) Importance for my work
(b) Importance for the patient

Finally, only for the healthcare workers three specific questions and a comment box were added:

1. Have a storage room for medical aids and materials (gauze, bandages, disinfectants…) at the beginning of the ward (Importance for my work activity).
2. Have a storage room for medical equipment and materials (gauze, bandages, disinfectants…) in the middle of the ward (Importance for my work activity).
3. Have a confined area for large equipment on the ward (Importance for my work activity).

The questionnaire was administered in a way that ensured respondents' anonymity, reducing the likelihood of social desirability bias. As mentioned above, participation was entirely voluntary, helping to mitigate response pressure and allowing individuals to provide honest feedback.

To conclude, the survey was uploaded into the online program Google Forms® and submitted to all employees with a request to participate in the study.

### 2.4 Data collection

The questionnaire was written and uploaded in the online survey programme Google Forms. A link was then sent to all employees with a request to fill it out. All the questionnaires were collected by the Medical Management and entered into a Microsoft Excel ® database. Trough preliminary descriptive statistics, they were inserted into reports, to be presented during a meeting held with the nursing coordinators of the wards involved.

### 2.5 Data analysis

All answers to the questions in order to be better understood have been represented in graphic form. In order to facilitate comprehension, the data relating to answers 4 and 5 are grouped with “HIGH” importance, 3 as “MEDIUM”, while 1 and 2 as “LOW” importance.

As explained above, the intention of this study was to analyse the quantitative data also by examining the data broken down according to variables that can influence the importance that certain items have on the perception of the environment.

This analysis led to the use of descriptive statistics to explore and compare the opinions of both patients and healthcare operators. The results highlighted key differences and similarities in their perceptions of the built environment, with particular focus on priority themes such as comfort and privacy.

## 3 Results

### 3.1 Patients results

The survey has been submitted to all the 927 patients. 521 questionnaires were collected with a response rate of 56%, completed on a voluntary basis.

In 89% of the cases the questionnaire was completed directly by the patient, in the remaining cases by a companion.

Within the patient population sample 55.1% were women and 44.9% men with an average age of 59 years.

75% of the patients came from Northern Italian regions (62% from Lombardy), 6% from Central Italian regions, 19% from Southern Italian regions.

In relation to the items, stands out the fact that the possibility of having a single room at one's disposal appears to be of no particular interest for patients, as the distribution of responses is slightly skewed towards indifference: 29% of the patients has reported that having a single room is of medium importance, 23% low and 19% high.

Similarly, on the whole, the choice of color for the wall coverings in the patient rooms is not of particular interest, although a substantial preference emerges for soft, uniform colors: less enthusiasm, in fact, was shown by the users regarding the possibility of using bright colors and/or landscapes: the possibility of having bright colored walls results to be not important at all for 49% of the patients, as well as the option of decorating with natural landscapes is not relevant for 38% of the patients. The section on entertainment devices shows a substantial appreciation (29% of patients reported high importance), which clearly points to the need to equip each workstation with Wi-Fi, satellite TV so that it can be enjoyed without disturbing the other patients.

The results and response rates are shown in Table 2.

**Table 2:** Survey results and response rates.

N°	Questions	Importance rate	Patient		Healthcare workers					
					Importance for my work			Importance for the patient		
					Medical director	Admin staff	Healthcare staff	Medical director	Admin staff	Healthcare staff
1	Have a single room, so that privacy is fully respected.	High	162	31%	56%	48%	51%	69%	67%	83%
		Medium	151	29%	19%	24%	29%	29%	21%	13%
		Low	198	38%	25%	27%	20%	2%	12%	4%
2	Have a two-bed room, so they can socialise to avoid the boredom of hospitalisation, and facilitate supervision.	High	177	34%	40%	48%	43%	46%	61%	61%
		Medium	182	35%	26%	18%	33%	32%	21%	30%
		Low	162	31%	34%	33%	25%	22%	18%	9%
3	In the case of a double room, how important is it to have a separation between the two beds by means of a curtain.	High	141	27%	64%	58%	72%	75%	73%	80%
		Medium	125	24%	13%	9%	15%	20%	18%	13%
		Low	261	50%	23%	33%	13%	5%	9%	6%
4	In the case of a double room, how important is the presence of a physical separation between the two beds through a movable wall.	High	99	19%	55%	48%	61%	64%	64%	73%
		Medium	109	21%	15%	12%	20%	22%	9%	16%
		Low	313	60%	30%	39%	18%	14%	27%	11%
5	Having a living area in the patient room so that the patient can relax without staying in bed.	High	234	45%	52%	42%	54%	82%	82%	86%
		Medium	167	32%	21%	30%	22%	9%	15%	10%
		Low	120	23%	27%	27%	24%	9%	3%	4%
6	Having the possibility for the patient to look out of the window while remaining in bed.	High	255	49%	45%	55%	48%	87%	76%	88%
		Medium	146	28%	26%	18%	27%	12%	18%	8%
		Low	115	22%	29%	27%	25%	1%	6%	4%
7	Have the windows of the in-patient rooms with a view of the surrounding nature (if the facility is located in the countryside) or of the surrounding buildings (if the facility is located in a city context).	High	234	45%	57%	64%	52%	91%	88%	87%
		Medium	151	29%	11%	9%	23%	7%	12%	7%
		Low	135	26%	32%	27%	25%	2%	0%	6%
8	Have an air-conditioning system in the room that the patient can regulate from their bed.	High	234	45%	52%	64%	63%	84%	91%	85%
		Medium	167	32%	24%	9%	22%	11%	3%	10%
		Low	120	23%	24%	27%	14%	5%	6%	5%
9	Having the possibility for the patient to adjust the room lighting from their bed.	High	313	60%	66%	70%	70%	98%	94%	94%
		Medium	151	29%	15%	9%	17%	2%	3%	6%
		Low	52	10%	19%	21%	13%	0%	3%	1%
10	Have a nurse call system accessible to the patient not only from the bed, but in every area of the room.	High	240	46%	68%	58%	78%	88%	82%	88%
		Medium	156	30%	14%	6%	13%	9%	15%	11%
		Low	125	24%	18%	36%	9%	3%	3%	1%
11	Provide a second bed or a bed chair for a possible patient partner/parent.	High	250	48%	48%	48%	57%	85%	91%	85%
		Medium	177	34%	20%	15%	25%	10%	9%	11%
		Low	94	18%	32%	36%	18%	5%	0%	4%
12	Positioning the bed in line with the door, so that it is also visible from the corridor and facilitates surveillance by the operators.	High	135	26%	65%	48%	72%	64%	64%	63%
		Medium	167	32%	15%	15%	13%	21%	24%	24%
		Low	224	43%	20%	36%	14%	15%	12%	14%
13	Presence of a video surveillance system connected to the guardhouse.	High	120	23%	67%	61%	79%	67%	67%	69%
		Medium	177	34%	15%	9%	11%	20%	27%	19%
		Low	219	42%	18%	30%	10%	13%	6%	12%
14	Have a private bathroom for the patient accessible from the room.	High	391	75%	70%	67%	82%	98%	97%	98%
		Medium	94	18%	9%	6%	10%	2%	3%	2%
		Low	31	6%	21%	27%	7%	0%	0%	0%
15	Have a bidet in the bathroom.	High	412	79%	49%	42%	66%	87%	91%	95%
		Medium	99	19%	19%	21%	15%	11%	3%	4%
		Low	31	6%	32%	36%	19%	2%	6%	2%
16	Room walls in soft, uniform colours.	High	188	36%	47%	55%	49%	60%	70%	73%
		Medium	162	31%	23%	21%	31%	25%	21%	20%
		Low	172	33%	30%	24%	20%	14%	9%	7%
17	Brightly coloured room walls.	High	52	10%	-	-	-	-	-	-
		Medium	89	17%	-	-	-	-	-	-
		Low	375	72%	-	-	-	-	-	-
18	Room walls decorated with natural landscapes, parks, mountain views, etc.	High	94	18%	-	-	-	-	-	-
		Medium	120	23%	-	-	-	-	-	-
		Low	307	59%	-	-	-	-	-	-
19	Presence in the room of services such as Wi-Fi connection, satellite TV.	High	328	63%	59%	73%	61%	90%	85%	97%
		Medium	120	23%	15%	9%	22%	10%	12%	3%
		Low	89	17%	25%	18%	17%	0%	3%	0%
20	In the case of double rooms, a dedicated TV in each patient bed equipped with headphones.	High	292	56%	-	-	-	-	-	-
		Medium	120	23%	-	-	-	-	-	-
		Low	135	26%	-	-	-	-	-	-
21	Presence of soundproofing in the room to exclude any noise from the ward corridor or neighbouring rooms.	High	203	39%	49%	45%	43%	73%	70%	76%
		Medium	162	31%	18%	30%	25%	19%	24%	14%
		Low	156	30%	33%	24%	32%	9%	6%	9%
22	Possibility for patients to be able to customise the furniture in their rooms by making small changes and/or enriching them with their own items.	High	42	8%	9%	33%	20%	26%	33%	50%
		Medium	94	18%	25%	21%	22%	34%	30%	22%
		Low	386	74%	66%	45%	58%	40%	36%	28%
23	Having the nursing room at the beginning of the ward.	High	-	-	23%	30%	23%	13%	45%	21%
		Medium	-	-	32%	27%	28%	43%	21%	35%
		Low	-	-	45%	42%	49%	44%	33%	44%
24	Having the nursing room in the centre of the ward.	High	-	-	46%	45%	73%	49%	55%	68%
		Medium	-	-	29%	15%	17%	27%	21%	21%
		Low	-	-	25%	39%	11%	23%	24%	10%

For a full view of response (writing format) please see the questionnaire and the comments reported as additional material.

### 3.2 Health professionals results

The ages of the participants were divided into four generations: GenZ (12–27 years), Millennials (28–44 years), Generation X (45–59 years), and Baby Boomers (>60 years), with the majority represented by Generation X (48%).

The majority of operators have significant seniority, with 24% of participants having worked for more than 30 years (52% >20 years).

48% of the operators have worked exclusively in INT, a percentage that increases to 58% when considering employees with 90% or more seniority in INT.

38% of the respondents are nurses, showing greater sensitivity than other roles. Overall, 57% of the respondents belong to the healthcare sector, while 29% are from the management world.

With regard to having a single room, so as to have full respect for privacy, the majority of doctors consider this to be of great relevance for their practice (56%).

For the two-bed room, the ratings do not show a clear preponderance of one response (high importance 42% for caregiver and 57% for patient). In the case of separation, the curtain is preferred over the movable wall (68% vs. 58%) with slightly higher values for nursing staff.

Among the elements considered to be of high importance for the patient that are also correlated with high importance for work are:

- Having the possibility for the patient to adjust the lighting of the room from their bed (95% high importance for the patient and 68% for health professionals).
- Having a nurse call system accessible to the patient not only from the bed, but in every area of the room (87% high importance for the patient and 73% for health professionals).
- To have a second bed or a bed chair for a possible carer/patient relative (86% high importance for the patient and 54% for health professionals).
- Have a private bathroom for the patient accessible from the room (98% high importance for the patient and 77% for health professionals).
- Having a bidet in the bathroom (92% high importance for the patient and 59% for the work activity).
- Presence in the room of services such as Wi-Fi Connection, Satellite TV (92% high importance for the patient and 59% for health professionals).

The results and response rated are shown in Table 2.

## 4 Discussion

### 4.1 Single vs. multiple patient room

Design choices concerning healthcare facilities must take into account not only technical-economic constraints and hygienic-organizational requirements, but also the preferences expressed by the end users: patients and operators. The socio-cultural context in which a structure is built, in fact, can be a determining factor in some design choices, leading to a certain difficulty in identifying a “universal” model that is applicable in all healthcare facilities, regardless of the type of welfare that supports them. Despite the responses of the patient sample there is a growing evidence that single hospital rooms improve patient dignity and privacy and reduce infection transmission (3). Perhaps the most striking fact to emerge from this survey is the substantial neutrality of the patient with respect to the choice of a single room as opposed to a double room: while to an outside eye the preference for a single room might in fact seem obvious, the patients themselves, and here in particular the cancer patients, do not attribute the same value to privacy, seeking instead the possibility of guaranteeing a minimum level of sociality: this importance was also confirmed during the first discussion with the operators, where the constant effort to place patients of a similar age group in the rooms in order to make the socialization experience easier emerged clearly.

### 4.2 Similarities and mismatches

The overall Table used for data collection appeared to be an effective option to compare the importance rate of patients and health professionals, for the whole list of items. The results from the two respondents are compared to provide a picture of the way patients and staff do or do not provide the same responses. The study detected a significant mismatch between the feedback: in 50% of the items the patients point of view didn't correspond to the staff preferences.

Concerning the possibility of having a single room while for the 76% of the healthcare professionals it is very important, it has been detected very low rate between patients (31%).

Even if literature states that a window view can promote positive moods or reduce stress (13), only the 45% of patients reported a high importance of having a window with a view, while the 89% of the healthcare professionals.

On the other hand, there is a notable extent of agreement between respondents about the importance of having a private bathroom and a bidet. For more than 75% of both respondents these elements resulted to be very important. Empowering patients with options in the treatment space, including capacity to control ambient have the potential to support treatment and empower patients (15). In this context, for the possibility of adjusting the room lighting from the bed, responses rates for a high importance were 60% between patients and 96% between healthcare professionals. Another relevant fairly common concern regards the presence in the room of services such as Wi-Fi connection and satellite TV: 63% of the patients and 93% of the healthcare workers reported a high rate of importance for it.

Lastly, a supportive amount of evidence encourages the importance of users involvement in programming and designing cancer centers (25). For this reason, it is important to point out that in this study for the 25% of the cases, the opinion of staff (considering the patient, not their work activity) does not correspond to the patient's actual point of view.

### 4.3 Education and multidisciplinarity

Another important aspect which emerged from the evaluation of the answers concerning the interior decoration choices, is that the general population is not sufficiently prepared to grasp the importance of the care environment as an element of care itself, to the point of pushing some patients to consider these aspects more related to tourist standards than to hospital standards.

Comparison and benchmarking with theoretical background and current practices leads in most cases to confirmation of the contents: in a few items, however, a deviation from the patients' perception is observed (single vs. double room, bathroom with/without bidet, personalization) or in the light of the operators' observations (room size, windows, video surveillance system and bedhead position) (26). The perception regarding the overall layout of the room (single vs. double) leads one to believe that there is no single model applicable on the facility, and the operators confirm this: in this regard, it is appropriate to bear in mind a possible bias linked to the type of target patients, a population characterized by a particularly critical psychological impact due to the base pathology, and in any case different from a non-oncological acute patient.

Patients represent an important voice in guiding the choices: their point of view may however be completely different from that of the planners and health management; in this sense, it could be useful in the case of a renovation and/or new building intervention, a form of consultation with the patients on the type and forecast of the intervention, which can then be mediated by the operators and the health management and thus support the design choices.

Through this study, the authors state that a joint effort by healthcare practitioners, healthcare architects and hospital managers is needed for the improvement and dissemination of further EBD research in healthcare, in particular in Italy where it is not widely used (27–29).

## 5 Conclusion

The present study investigated the insights of oncological patients and healthcare professionals about certain aspects of inpatient rooms at the INT. The choice of items and features explored during the research and survey activity refer to the scientific literature consulted.

The mixed methodology used in the study allowed to obtain information on the relationship between the physical spatial characteristics of inpatient rooms and the perceptions of the users. The findings brought to light, in most cases, a mismatch between patients and staff views about the importance of presence/absence of determinate elements. The importance rate for patient and for staff matched in few cases, for example about having a two-bed room which resulted to be indifferent for both, or about having a private bathroom which turned out to be very important to almost everyone.

The study provides implementation in research coming up with new insights into the relation between the built environment and the oncological patients perception. To address the issues identifies it is recommended to involve patients and healthcare professionals during the design phase in order to meet the final objective of integrating these preferences into planning process. To address this some practical approaches are proposed, such as: co-design workshops, which could bridge the gap between professional design expertise and user experience; patient advisory panels, who could review design plans and provide insights; pilot testing of spaces, as implementing prototype rooms where both healthcare operators and patients can provide real-time feedback could help refine the final project to better meet their needs.

In conclusion, the findings could have an impact on society, considering it could lead to an improvement in care delivery in the oncological context.

## 6 Limitations and future developments

However, there are few limitations to be mentioned such as: the sample of participants might not be representative of all categories of workers in the hospital, as some operating units might have been excluded for certain profiles. There might also be limitations related to the generalization of the results, as the users' preferences and needs might vary according to the geographical, cultural and institutional context.

Starting from these considerations, future developments of the study include exploring the sample, scaling up the survey in different hospitals and using the results of the study to develop design guidelines for hospital environments that can be used by designers and managers of healthcare facilities. This would enable guidance on the design and refurbishment of hospital spaces oriented towards maximizing user well-being and satisfaction, matching the preferences of patients, doctors and nurses in hospital environments.

Moreover, since the preferences may shift over time, it would be interesting to explore them over the course of treatment, in order to distinguish, for example, preferences of diagnosed patients and of patients undergoing long-term treatment.

In this context, an analysis on how the design elements influence health outcomes, such as recovery rates, would increase value to the research.

In conclusion, future research should also explore the practical implications of healthcare professionals' preferences for single-patient rooms. A cost-benefit comparing different room configurations could provide valuable insights for hospital planning and policy decisions.

## Data Availability

The original contributions presented in the study are included in the article/Supplementary Material, further inquiries can be directed to the corresponding author/s.
