# Supplementary material for: Healthcare workers and adult patients preferences of hospital built environment. Survey in ordinary surgery and medical oncology ward at the Italian National Oncology Institute
Source: Front Health Serv. 2025 Apr 7;5:1546103. doi: 10.3389/frhs.2025.1546103 (PMC12010251; doi:10.3389/frhs.2025.1546103)
Supplement: Supplementary file 1 [file Datasheet1.pdf]

SUPPLEMENTARY MATERIAL – DETAILS QUESTIONNAIRE ON THE SAMPLE OF HEALTH PROFESSIONALS

In order to allow for any in-depth analysis or quantitative statistical analysis, biographical elements and more details on the work experience of the respondent were collected, such as Role, Department, Gender and Age. Subsequently grouped by age group:

- <28 (GenZ)
- 28-44 (Millennials)
- 45-59 (X Generation)
- >59 (Baby Boomers)

Years of service and successively grouped by year bands:

- 0-5
- 6-10
- 11-15
- 16-20
- 21-25
- 26-30
- >30

Years of service in INT and structure membership.
From which department membership was deduced:

- Department of Oncological Surgery
- Services and Advanced Diagnostics Department
- Diagnostic Imaging and Radiotherapy Department
- Oncology and Oncohaematology Department
- Experimental Oncology Department
- Administrative Management
- General Management
- Health Management
- Other

Subsequently grouped into:

- Department of Oncology and Oncohaematology
- Department of Oncological Surgery
- Other

The conformation of the inpatient room in the department was deduced:

- Single Room
- Double room
- Triple room
- No room

The average length of stay in the ward was deduced and then grouped into:

- Day service (0-1 days)
- Short stays (2-4 days)
- Long hospital stays (>5 days)
