# Supplementary material for: Healthcare workers and adult patients preferences of hospital built environment. Survey in ordinary surgery and medical oncology ward at the Italian National Oncology Institute
Source: Front Health Serv. 2025 Apr 7;5:1546103. doi: 10.3389/frhs.2025.1546103 (PMC12010251; doi:10.3389/frhs.2025.1546103)
Supplement: Supplementary file 2 [file Datasheet2.pdf]

SUPPLEMENTARY MATERIAL – THE QUESTIONNAIRE

	Not important	Quite important	Very important
**1.** Have a single room, so that privacy is fully respected.	1 2 3 4 5		
2. Have a two-bed room, so they can socialise to avoid the boredom of hospitalisation, and facilitate supervision.	1 2 3 4 5		
3. In the case of a double room, how important is it to have a separation between the two beds by means of a curtain.	1 2 3 4 5		
**4.** In the case of a double room, how important is the presence of a physical separation between the two beds through a movable wall.	1 2 3 4 5		
5. Having a living area in the patient room so that the patient can relax without staying in bed.	1 2 3 4 5		
6. Having the possibility for the patient to look out of the window while remaining in bed.	1 2 3 4 5		
**7.** Have the windows of the in-patient rooms with a view of the surrounding nature (if the facility is located in the countryside) or of the surrounding buildings (if the facility is located in a city context).	1 2 3 4 5		
8. Have an air-conditioning system in the room that the patient can regulate from their bed.	1 2 3 4 5		
9. Having the possibility for the patient to adjust the room lighting from their bed.	1 2 3 4 5		
10. Have a nurse call system accessible to the patient not only from the bed, but in every area of the room.	1 2 3 4 5		
11. Provide a second bed or a bed chair for a possible patient partner/parent.	1 2 3 4 5		
12. Positioning the bed in line with the door, so that it is also visible from the corridor and facilitates surveillance by the operators.	1 2 3 4 5		
13. Presence of a video surveillance system connected to the guardhouse.	1 2 3 4 5		

14. Have a private bathroom for the patient accessible from the room.	1 2 3 4 5
15. Have a bidet in the bathroom.	1 2 3 4 5
16. Room walls in soft, uniform colours.	1 2 3 4 5
17. Brightly coloured room walls.	1 2 3 4 5
18. Room walls decorated with natural landscapes, parks, mountain views, etc.	1 2 3 4 5
19. Presence in the room of services such as Wi-Fi connection, satellite TV.	1 2 3 4 5
20. In the case of double rooms, a dedicated TV in each patient bed equipped with headphones.	1 2 3 4 5
21. Presence of soundproofing in the room to exclude any noise from the ward corridor or neighbouring rooms.	1 2 3 4 5
22. Possibility for patients to be able to customise the furniture in their rooms by making small changes and/or enriching them with their own items.	1 2 3 4 5
23. Having the nursing room at the beginning of the ward.	1 2 3 4 5
24. Having the nursing room in the centre of the ward.	1 2 3 4 5

Fondazione IRCCS Istituto Nazionale dei Tumori — Sistema Socio Sanitario Regione Lombardia — POLITECNICO MILANO 1863 DIPARTIMENTO DI ARCHITETTURA, INGEGNERIA DELLE COSTRUZIONI E AMBIENTE COSTRUITO

Gentilissimo

la Direzione Medica di Presidio, in collaborazione con il Politecnico di Milano, sta avviando un'analisi su alcuni aspetti della struttura e degli ambienti ospedalieri, che si vorrebbe corrispondere al meglio alle esigenze e aspettative dei pazienti.
Il presente questionario, che è anonimo, è finalizzato a raccogliere le vs suggestioni e impressioni in modo da poter individuare le aree dove in fase di progettazione e realizzazione sarà necessario concentrare maggiormente i ns sforzi.
Per eventuali ulteriori informazioni potrà scrivere al seguente indirizzo mail: direttore.medico@istitutotumori.mi.it , buona compilazione!

Data della compilazione ___/___/____ Paziente |_| Accompagnatore |_|

Sesso M |_| F |_| Età |_|_| Provincia di provenienza ________________________

In una scala da 1 a 5, dove 1 corrisponde a "per nulla importante, indifferente" e 5 è "molto importante, fondamentale" la preghiamo di valutare, apponendo una X, le seguenti tematiche:

	Per nulla Importante		***Abbastanza Importante***		***Molto Importante***
1-Avere una camera singola, in modo da avere un pieno rispetto della privacy	1	2	3	4	5
2-Avere una camera a due letti, in modo da poter socializzare per evitare la noia della degenza, e facilitare la vigilanza	1	2	3	4	5
3-In caso di camera doppia, presenza di una separazione tra i due posti letto attraverso una tenda	1	2	3	4	5
4-In caso di camera doppia, presenza di una separazione fisica tra i due posti letto attraverso una parete mobile	1	2	3	4	5
5-Poter disporre di una zona giorno nella camera di degenza in modo da potersi rilassare senza restare a letto	1	2	3	4	5
6-Avere la possibilità di guardare al di fuori della finestra restando a letto	1	2	3	4	5
7-Avere una vista sulla natura circostante (se la struttura è ubicata nel verde) o sugli edifici circostanti (se la struttura è collocata in un contesto cittadino)	1	2	3	4	5
8-Disporre di un impianto di climatizzazione della camera regolabile anche dal letto	1	2	3	4	5

FONDAZIONE IRCCS ISTITUTO NAZIONALE DEI TUMORI
Istituto di Ricovero e Cura a Carattere Scientifico di diritto pubblico
C.F. 80018230153 - P. IVA 04376350155
Via Venezian, 1 - 20133, MILANO - Tel. 02/23901
www.istitutotumori.mi.it

	Per nulla Importante		Abbastanza Importante		Molto Importante
9-Avere la possibilità di regolare l'illuminazione della camera anche dal letto	1	2	3	4	5
10-Disporre di un impianto di chiamata infermieri accessibile non solo dal letto, ma in ogni area della camera	1	2	3	4	5
11-Disporre di un secondo letto oppure di una poltrona letto per un eventuale accompagnatore/parente	1	2	3	4	5
12-Posizionamento del letto in linea con la porta, in modo che sia visibile anche dal corridoio e favorire la sorveglianza da parte degli operatori	1	2	3	4	5
13-Presenza di un sistema di videosorveglianza collegato alla guardiola	1	2	3	4	5
14-Disporre di un bagno riservato accessibile dalla camera	1	2	3	4	5
15-Disporre, all'interno del bagno, di un bidet	1	2	3	4	5
16-Pareti della camera di colori tenui e uniformi	1	2	3	4	5
17-Pareti della camera di colori vivaci e accesi	1	2	3	4	5
18-Pareti della camera decorate con paesaggi naturali, parchi, vedute montane ecc.	1	2	3	4	5
19-Presenza nella stanza di servizi quali Connessione Wi-Fi, Tv Satellitare	1	2	3	4	5
20-In caso di camera doppia, una TV dedicata ad ogni letto dotata di cuffie	1	2	3	4	5
21-Presenza di una insonorizzazione della camera tale da escludere qualsiasi rumore dal corridoio del reparto o dalle camere vicine	1	2	3	4	5
22-Riterrebbe utile poter personalizzare l'arredo della camera attraverso piccoli spostamenti e/o arricchendolo con suppellettili proprie	1	2	3	4	5

Eventuali commenti/suggerimenti: ____
____
____
____

GRAZIE PER LA COLLABORAZIONE!
