# Supplementary material for: Healthcare workers and adult patients preferences of hospital built environment. Survey in ordinary surgery and medical oncology ward at the Italian National Oncology Institute
Source: Front Health Serv. 2025 Apr 7;5:1546103. doi: 10.3389/frhs.2025.1546103 (PMC12010251; doi:10.3389/frhs.2025.1546103)
Supplement: Supplementary file 3 [file Datasheet3.pdf]

SUPPLEMENTARY MATERIAL – PATIENTS' COMMENTS

Below are reported the comments that patients felt to add by completing the final part of the questionnaire: the comments are faithfully reproduced from the original, also from the graphic point of view.

ADJUSTABLE AIR CONDITIONING IN EACH ROOM.

The single room is undoubtedly very comfortable, but I like to socialise. A second person can also be helpful. I propose pairing the patients by age group.

Have a common room in the ward where you can socialise without a TV but with suitable tools such as photographs to look at and discuss or sentences on the walls and chairs placed so that you can look each other in the face.

I describe below further elements that I consider important: cleanliness of room and bathroom, air quality and recirculation of suction in the bathroom to eliminate unpleasant odours. The furniture, bathrooms, bedside tables, larger beds should be redone.

Put up partitions between the beds, new blankets, personal TV, ducts out of sight, curtains in the windows, even if the important thing is doctors.

EDUCATION, RESPECT FOR ELDERLY PATIENTS (ABOVE ALL), CAMERAS
WARM THANKS TO ALL THE STAFF

Nobody, it's already going very well. Everything is more than perfect. Thank you!

Change the worn-out blankets. Have a mini library and not outside the ward.

Renovate the facility a bit in general. Room 8 third floor F.

FURNISHINGS WITH A MORE TASTEFUL FINISH REMINISCENT OF HOME ENVIRONMENTS

Provide blankets that are not crumpled.

ROOM DOOR OPEN AT ALL TIMES, SMOKERS' AREA LIVEABLE, AND AREA FOR ANY RELATED PROBLEMS

Wider toilet and bidet spacing and higher toilets and more window insulation to avoid too much cold or heat.

DOESN'T THIS SOUND MORE LIKE A HOLIDAY HOTEL THAN A HOSPITAL?

WE WOULD ALL LIKE TO HAVE EVERYTHING... BUT THE COSTS? A MADNESS! (FROM RSM)

For me it is important the availability the professionalism and humanity that I found.

Larger bathrooms would be desirable is very difficult to enter with floor lamps, IVs, etc.

I only ask for a larger bathroom and preferable shower for the bathroom in the room instead of the tub.

Everything OK.

Congratulations to the staff of this ward for their work and their patience and kindness for us patients...especially with me. Congratulations again.

In the room for two better with roommates of the same age.

CUPBOARD WITH SHELVES AND BIGGER BED.

Priority: comfortable bathroom accessible with IV stands or wheelchair.

Reconstruction of a new facility in a more accessible area surrounded by nature (urban green). See Versilia Hospital project. Arc. Zambelli.

Only positive comments for the Istituto Tumori di Milano. Kind and cheerful professional staff (with us patients at least).

MANOEUVRABLE OPENING/CLOSING SYSTEM.

point 3 and 4 should be evaluated by the medical staff according to severity and age

being in a room with people of a similar age.

I CONSIDER A FEW EXTRA SHELVES USEFUL TO BE ABLE TO PLACE MEDICAL AIDS FOR DAILY USE.

We would like a plug near the bedside table for charging mobile phones, we have one in the room for 2 people and away from the bed.

Remove the wardrobe divider.

Useful safe (present).

The possibility of having a room/lounge where we could stay with relatives.

Congratulations to all nursing and care staff for their professionalism and kindness. ‘TOP’ medical staff. Only criticism: non-existent room cleaning.

More wall plugs, larger lockers.

Extend visiting hours, especially on the day of surgery, to several visitors with alternating access, including spaces outside the room.

The aspect I consider most important concerns the staff, feeling welcome and cared for. Thank you.

Parking spaces to facilitate access to the facility and no charge. It's a public service for sick people who shouldn't have to worry about parking or paying. p.s. Friendly helpful staff - very good - rating 5.

A bigger bathroom and a bigger wardrobe.

I have no problem adjusting to whatever conditions exist I hope not to have long stays.

1 Ceilings need to be coloured not so much the walls. 2 Rounded edges floor wall. 3 More electrical plugs accessible from the bed.

Intercom to communicate in case of isolation during a relative's visit.

I would recommend an establishment where the connections to car parking spaces are adequate or even in excess in view of the development of the building.

-No 1.5 L water bottles (but 0.5 L bottles) -Water with a raised board -Lighter chairs, too heavy -No 3-bed rooms and 1 bathroom.

All the proposals you implement would totally change the guests' stay and make the illness short and effective with a speedy recovery.

Thank you very much for your competence, kindness and helpfulness.

TV in the room would be nice!

Bench before entering the ward while waiting for admission.

You don't need 2 or 3 televisions important 1 so the day passes more quickly

A kitchen in the ward.
